# The Effects of Cocaine on Different Redox Forms of Cysteine and Homocysteine, and on Labile, Reduced Sulfur in the Rat Plasma Following Active versus Passive Drug Injections

**DOI:** 10.1007/s12640-013-9403-6

**Published:** 2013-05-16

**Authors:** Danuta Kowalczyk-Pachel, Grażyna Chwatko, Małgorzata Iciek, Joanna Czyżyk, Małgorzata Filip, Lidia Włodek, Elżbieta Lorenc-Koci

**Affiliations:** 1 The Chair of Medical Biochemistry, Jagiellonian University Collegium Medicum, 7, Kopernika St., 31-034 Kraków, Poland; 2 Department of Environmental Chemistry, University of Łódź, 163, Pomorska St., 90-236 Łódź, Poland; 3 Laboratory of Drug Addiction Pharmacology, Institute of Pharmacology, Polish Academy of Sciences, 12, Smętna St., 31-343 Kraków, Poland; 4 Department of Neuro-Psychopharmacology, Institute of Pharmacology, Polish Academy of Sciences, 12, Smętna St., 31-343 Kraków, Poland; 5 Department of Toxicology, Faculty of Pharmacy, Jagiellonian University College of Medicine, Medyczna 9, 30-688 Kraków, Poland

**Keywords:** Cocaine, Cysteine, Homocysteine, Self-administration, Plasma, Yoked procedure

## Abstract

The aim of the present studies was to evaluate cocaine-induced changes in the concentrations of different redox forms of cysteine (Cys) and homocysteine (Hcy), and products of anaerobic Cys metabolism, i.e., labile, reduced sulfur (LS) in the rat plasma. The above-mentioned parameters were determined after i.p. acute and subchronic cocaine treatment as well as following i.v. cocaine self-administration using the yoked procedure. Additionally, Cys, Hcy, and LS levels were measured during the 10-day extinction training in rats that underwent i.v. cocaine administration. Acute i.p. cocaine treatment increased the total and protein-bound Hcy contents, decreased LS, and did not change the concentrations of Cys fractions in the rat plasma. In turn, subchronic i.p. cocaine administration significantly increased free Hcy and lowered the total and protein-bound Cys concentrations while LS level was unchanged. Cocaine self-administration enhanced the total and protein-bound Hcy levels, decreased LS content, and did not affect the Cys fractions. On the other hand, yoked cocaine infusions did not alter the concentration of Hcy fractions while decreased the total and protein-bound Cys and LS content. This extinction training resulted in the lack of changes in the examined parameters in rats with a history of cocaine self-administration while in the yoked cocaine group an increase in the plasma free Cys fraction and LS was seen. Our results demonstrate for the first time that cocaine does evoke significant changes in homeostasis of thiol amino acids Cys and Hcy, and in some products of anaerobic Cys metabolism, which are dependent on the way of cocaine administration.

## Introduction

Cocaine is an alkaloid found in the leaves of the South American plant *Erytroxylon coca*. It is one of the most addictive substances for humans and animals (Evans 1981). Despite the unceasing research aimed to explain the cocaine actions, its contribution to health disturbances is still insufficiently understood especially the drug intake-related death cases have not been satisfactorily explained. For the latter reasons, it is necessary to elucidate the entirety of pathogenic action of this drug of abuse on the whole human and animal organisms.

It has been established that pharmacological action of cocaine (increases in dopamine neurotransmission) and its biodegradation in mammalian organisms is associated with the oxidative stress (Dietrich et al. 2005; Visalli et al. 2005). Although, the nontoxic hydrolysis is a major cocaine metabolic pathway, oxidative biotransformation catalyzed by microsomal enzymes does occur, and leads to the formation of “reactive metabolites” that can generate reactive oxygen species (ROS) by redox cycling (Kovacic 2005; Visalli et al. 2005). The later observation suggests that the ROS generation may be implicated in cocaine intoxication and addiction.

A natural reservoir of reductive capacity of cells and plasma is primarily dependent on nonprotein (NPSH) and protein thiol compounds that are responsible for maintaining the physiological intra- and extra-cellular thiol redox buffer (Kemp et al. 2008). On the other hand, in pathological conditions, the oxidative stress elicits the disturbances in redox potential (Kemp et al. 2008). In consequence, the ensuing changes in concentrations of different redox forms of thiols in cells and plasma lead to disturbances in the redox-mediated signal transduction pathways of many biological processes (Forman et al. 2004, 2008). In this context, the tripeptide glutathione (GSH), cysteine (Cys), and homocysteine (Hcy) seem to be important.

Cocaine administration decreased the blood concentration of the main cellular antioxidant GSH (Visalli et al. 2005; Labib et al. 2001, 2002), and the effect was suggested to be a result of the increased production of ROS by this drug of abuse (Dietrich et al. 2005; Kovacic 2005; Visalli et al. 2005). In contrast, in the liver, cocaine increased the GSH concentration (Labib et al. 2001, 2002), which may be explained by compensatory de novo synthesis of this antioxidant in hepatocytes (Wiener and Reith 1990; Mehanny and Abdel-Rahman 1991). Interestingly, in brain, the cocaine treatment induced a decline of GSH content in the hippocampus (Muriach et al. 2010), but no alterations were observed in the prefrontal cortex and striatum (Wiener and Reith 1990). Moreover, it was reported that in the nucleus accumbens, cocaine inhibited activity of the *x*_c_^−^ transport system, highly specific for cystine (CySS) and glutamate (Baker et al. 2002, 2003; Madayag et al. 2007; Kau et al. 2008). This anionic amino acid transporter localized in astrocyte plasma membrane catalyzes the Na^+^-independent exchange of the extracellular CySS for intracellular glutamate in a 1:1 stoichiometric ratio (McBean 2002). In the cell cytosol, CySS is rapidly reduced to Cys that is used either for proteins or de novo GSH synthesis (Meister and Anderson 1983). On the other hand, a growing body of evidence demonstrated that the system *x*_c_^−^ might act on its own as a GSH-independent redox cycle over the plasma membrane (Conrad and Sato 2012). Hallmarks of this cycle include: CySS uptake; intracellular reduction to Cys, and secretion of Cys excess to the extracellular space. The enhanced extracellular Cys levels provide a reducing microenvironment required for proper cell signaling and communication (Conrad and Sato 2012). Consistently, *N*-acetylcysteine (NAC), a Cys precursor, was observed to restore both CySS/glutamate exchanger activity and to produce a significant decline in cocaine-induced reinstatement in rats (Baker et al. 2003; Madayag et al. 2007; Zhou and Kalivas 2008; Kau et al. 2008; Amen et al. 2011; Kupchik et al. 2012), as well as reduced cocaine use, and craving in cocaine abusers (LaRowe et al. 2006; Mardikian et al. 2007; Amen et al. 2011). Until now, it is difficult to judge to what extent NAC acts as a Cys and GSH precursor, and to what extent as a thiol antioxidant directly affecting the thiol-disulfide balance displaced by cocaine. Furthermore, the Cys/CySS redox system is the largest pool of this low-molecular weight (LMW) thiol in plasma. An array of studies confirmed that the changes in the extracellular Cys/CySS ratio, by influencing the redox potential of plasma and cells, regulated the most important cellular processes, such as cell proliferation, differentiation, and apoptosis (Jones et al. 2004; Kemp et al. 2008). Interestingly, the extracellular Cys/CySS couple plays a key role in the regulation of early events of atherosclerosis and could be useful as a potential marker for some vascular diseases (Go and Jones 2005).

**Fig. 1:**
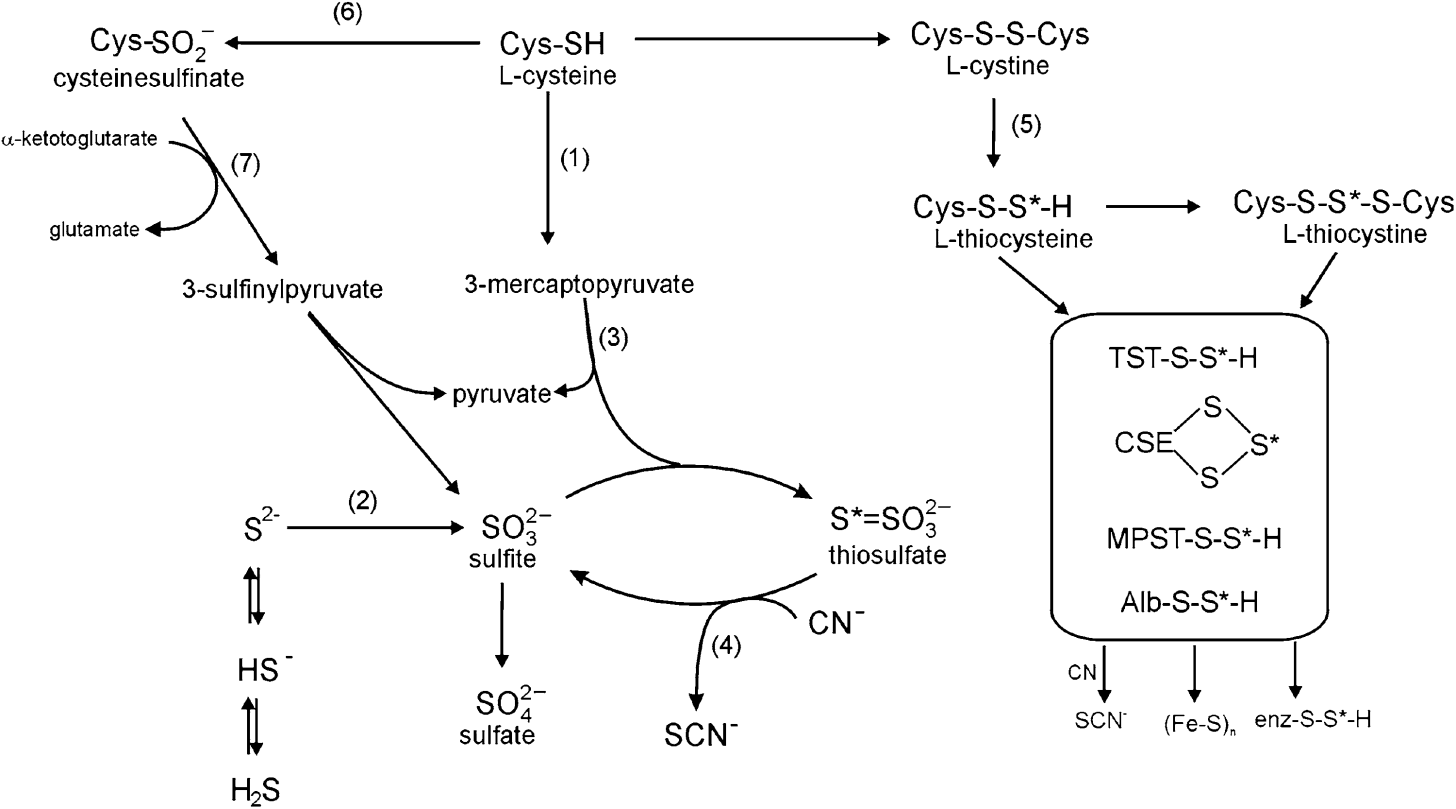
Aerobic and anaerobic metabolisms of cysteine. S*—sulfane sulfur, (1) cysteine aminotransferase, (2) nonenzymatic or catalyzed by sulfide oxidase, (3) 3-mercaptopyruvate sulfurtransferase (MPST), (4) rhodanese (TST), (5) γ-cystathionase (CSE), (6) cysteine dioxygenase, (7) aspartate aminotransferase, Alb: albumin, CN^−^: cyanide ion, and SCN^−^: thiocyanate ion

Moreover, it should be added that Cys sulfur can be metabolized either by aerobic or anaerobic route. The aerobic Cys metabolism yields sulfates and taurine in which sulfur atom has the highest (+6) oxidation state (Fig. 1; Cooper 1983). Anaerobic metabolism leads to biosynthesis of labile, reduced sulfur (LS) which has an oxidation state of 0 or −1 and is always bound with another sulfur atom (Fig. 1; Cooper 1983; Iciek and Włodek 2001). The products of anaerobic Cys metabolism comprise a pool of compounds bearing LS that show regulatory (Iciek and Włodek 2001; Toohey 2011) and antioxidant (Everett et al. 1994) properties (Fig. 1). Moreover, sulfur bound with proteins in the form of hydropersulfides is a direct precursor of hydrogen sulfide (H_2_S), the third of gaseous mediators, apart from nitric oxide (NO) and carbon oxide (CO), exhibiting vasorelaxant action (Chen et al. 2007). All the above observations appeared to suggest that cocaine could affect not only the physiological concentrations of different redox forms of Cys but also its anaerobic metabolism.

It is assumed that Hcy, another amino acid found in plasma, may be dysregulated by cocaine. It is well known that high level of Hcy, so-called hyperhomocysteinemia, is a phenomenon accompanying slow coronary flow (SCF) (Barutcu et al. 2005). Since cocaine users and addicts also suffer from SCF, caused by microvascular spasm with normally functioning coronary arteries (Turhan et al. 2007), it may be expected that cocaine can also cause changes in different redox forms of Hcy.

Thus, the pro-oxidant action of cocaine, and accompanying disturbances in GSH levels prompted us to investigate the cocaine effect on plasma levels of the remaining two thiol amino acids, i.e., Hcy and Cys (a glutathione precursor) as well as on products of anaerobic metabolism of cysteine sulfur (LS compounds) following cocaine administration in rats according to different schedules. These studies were conducted on rats receiving either acute or chronic intraperitoneal (i.p.) cocaine administration. Additionally, intravenous (i.v.) cocaine self-administration and 10-day extinction training with yoked procedure were examined. We expected to obtain the results that can shed a new light on the relationships between cocaine use and disturbances in plasma Cys, Hcy, and LS homeostasis.

## Materials and Methods

### Animals

Male Wistar rats (280–300 g), delivered by the licensed breeder (Charles River, Germany), were housed 4/cage or individually (self-administration) in standard plastic rodent cages in a colony room maintained at 20 ± 1 °C and at 40–50 % humidity under a 12-h light–dark cycle (lights on at 06:00). Animals had free access to standard animal food and water during the 7-day habituation period. Then, the rats used for the self-administration procedures were maintained on limited water during the initial training sessions (see below). All experiments were conducted during the light phase of the light–dark cycle (between 08:00 and 15:00) and were carried out in accordance with *the National Institutes of Health Guide for the Care and Use of Laboratory Animals*, and with approval of the Bioethics Commission as compliant with the Polish Law (21 August 1997). The animals were experimentally naive.

### Drugs

Cocaine hydrochloride (National Institute on Drug Abuse, RTI International, USA) was dissolved in sterile 0.9 % NaCl and given either i.v. (0.1 ml/infusion) or i.p. Control rats were administered solvent in the same way.

### Cocaine Self-Administration and Extinction Training

All rats used in these studies underwent the same training procedure and surgery. First, animals were trained to lever press in standard operant conditioning chambers (Med-Associates, USA) under a fixed ratio (FR) 5 schedule of water reinforcement which means that each 5 lever presses on the “active” lever resulted in delivery of one portion of water (Fijał et al. 2010). Then, the rats were implanted with catheters flushed every day with 0.1 ml of saline solution containing heparin (70 U/ml, Biochemie GmbH, Austria) and 0.1 ml of solution of cephazolin (10 mg/ml; Biochemie GmbH, Austria), as described previously (Fijał et al. 2010). Rats were allowed a 10-day recovery after surgical procedures before the start of the experiments. Later on, all animals were deprived of water for 18 h and trained in one 2-h session to press lever on an FR5 schedule for water reinforcement. Then, the animals were divided into two subgroups that began lever pressing for cocaine reinforcement during 2-h daily sessions performed 6 days/week (maintenance), and from that time they were given water ad libitum. Each completion of an FR5 schedule resulted in an infusion of cocaine (0.5 mg/kg over 5 s). A tone (2,000 Hz; 15 dB above ambient sound levels) and illumination of the stimulus light directly above the “active” lever were presented for 5 s, concurrent with a successful response for cocaine, following each injection there was a 20 s time-out period. Response on the “inactive” lever never resulted in cocaine delivery. An arbitrary acquisition criterion required that active lever presses vary by 10 % or less over three consecutive days during the maintenance.

One group of rats was sacrificed immediately following the last 2-h cocaine maintenance self-administration session following a 14-day series of cocaine self-administration, while another group underwent an extinction training period. During this extinction phase, the animals had 2-h daily training sessions with no delivery of cocaine or the presentation of the conditioned stimulus. On the 10th day of extinction, animals’ responses on the “active” lever fell to <10 % of the responses as the active lever reached during maintenance, and the rats were sacrificed immediately following the session while their brains were used for further biochemical assays.

### “Yoked” Protocol

Rats were tested simultaneously in groups of three with two rats serving as “yoked” controls that received an injection of saline or cocaine which was not contingent on responding, each time a response-contingent injection of 0.5 mg/kg cocaine was self-administered by the paired rat (Pomierny-Chamioło et al. 2012). Either cocaine or saline yoked injection was accompanied by the presentation of cue (tone and light). Unlike the self-administering rats, lever pressing by the “yoked” rats was recorded but had no programmed consequence. Yoked groups were sacrificed at the same time as rats self-administering cocaine or rats which underwent the extinction training.

### Acute or Subchronic Passive Cocaine Administration

Rats were given either acute or repeated (5 days) injections of cocaine (10 mg/kg) or vehicle in home cages.

### Collection of Blood Samples

The rats were killed by decapitation immediately after the termination of cocaine self-administration or its passive injection as well as after the last session of extinction training. In the case of i.p. cocaine injection, rats were killed 1 h after the first or last dose of this drug. Immediately after decapitation, the animals’ trunk blood was collected into tubes coated with EDTA. Blood samples were centrifuged at 2,000×*g* min, and plasma samples were collected.

Plasma thiols (Cys and Hcy) circulate both as protein-bound forms and free forms, including oxidized and reduced thiols. Therefore, different analytical steps were performed for the determination of total and free thiols as well as for LS. The content of protein-bound thiols for each plasma specimen was calculated as a difference between the total and free amounts.

### HPLC Measurements

The levels of total thiols, sulfide liberated by reduction and free thiols, were measured by HPLC after precolumn derivatization with 2-chloro-1-methylquinolinium tetrafluoroborate (CMQT) (Bald and Głowacki 2001), and separation and quantitation by ion-pairing reversed-phase liquid chromatography (Bald et al. 2004; Chwatko and Bald 2009).

### Determination of Total Thiols and Sulfide Liberated by Reduction

A 100 μl of plasma was mixed with 50 μl of 0.2 M phosphate buffer (pH 7.8) containing 2 mM EDTA, and 10 μl of 0.25 M tris(2-carboxyethyl)phosphine (TCEP). After a 15 min reduction at room temperature, 10 μl of 0.1 M CMQT was added, vortexed and kept at room temperature for 5 min, followed by the addition of 15 μl of 50 % perchloric acid (PCA) solution. Precipitated proteins were then removed by centrifugation at 12,000×*g* for 10 min, supernatant was transferred to a vial and injected into the HPLC system.

### Determination of Free Thiols

A 100 μl of plasma was mixed with 10 μl of 50 % PCA, vortexed and protein was separated by centrifugation (12,000×*g*, 10 min). The supernatant was decanted and alkalized to around pH 7 with 2.5 M sodium hydroxide. Next, 50 μl of 0.2 M phosphate buffer (pH 7.8) containing 2 mM EDTA, and 10 μl of 0.25 M TCEP were added and kept at room temperature for 15 min. Then, 10 μl of 0.1 M CMQT were added vortex-mixed and incubated at room temperature for 5 min, followed by addition of 15 μl of 50 % PCA. This solution was injected into the HPLC system.

### HPLC Analysis

The liquid chromatography equipment used for the analysis was manufactured by Hewlett-Packard (1100 Series system, Waldbronn, Germany), and consisted of a quaternary pump, autosampler, thermostated column compartment, vacuum degasser, and diode-array detector and controlled by an HP ChemStation software. For the pH measurement, an HI 221 (Hanna Instruments, Woonsocket, RI, USA) pH meter was used. Water was purified using a Millipore Milli-QRG system (Millipore, Vienna, Austria).

Final analytical solutions (20 μl) were injected into the Zorbax SB-C18 (150 × 4.6 mm, 5 μm) column (Agilent Technologies). For separation of 2-S-quinolinium derivatives of thiols from each other, and sulfide from reagent excess chromatographic condition described earlier (Bald et al. 2004; Chwatko and Bald 2009) were adopted with a slight modification. Briefly, the elution profile was as follows: 0–8 min, 10–35 % B; 8–10 min, 35–60 % B; 10–12 min, 60–10 % B; 12–13 min, 60 % B. Elution solvent (A) was 0.07 M trichloroacetic acid buffer (pH 1.6 prepared from 0.07 M TCA and 0.07 M LiOH) and (B) acetonitrile. The temperature was 25 °C, the flow-rate 1 ml/min and the detector wavelength 355 nm for thiols and 375 nm for sulfide. Identification of peaks was based on the comparison of retention times and diode-array spectra, taken at real time of analysis, with the corresponding set of data obtained for authentic compounds.

### Statistics

The significance of differences between the control group and that receiving i.p. cocaine (acutely or subchronically) was estimated by Student’s *t* test. A two-way ANOVA for repeated measures, and a one-way ANOVA, followed (if significant) by Tukey test were used for a statistical analysis of differences among yoked saline (YS), cocaine self-administration (SA), and yoked cocaine (YC) groups. A *p* value <0.05 was considered as statistically significant.

## Results

### Behavioral Studies

**Fig. 2:**
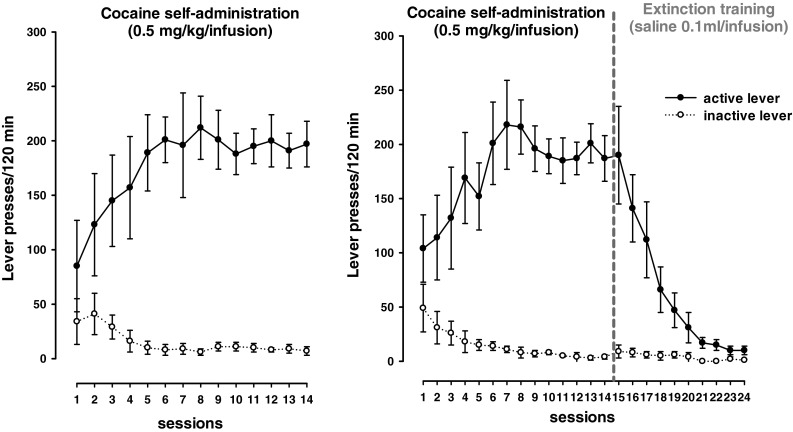
The number of active and inactive lever presses in rats that acquired and maintained cocaine (0.5 mg/kg/infusion) self-administration (*left panel*) and following 10-day extinction training (*right panel*). The number of animals per group, *n* = 10. Data are presented as the mean ± SEM, *** *p* < 0.001 versus inactive lever

After self-administration sessions, animals in two experimental groups showed stable lever-pressing rates during the last three self-administration days with less than a 10 % difference in their daily intake of cocaine (Fig. 2). The mean number of cocaine infusions per day during the last three self-administration days varied from 28 to 31. During 14 experimental sessions, animals received from 181 to 191 mg/kg of cocaine. Rats pressed significantly more frequently on the “active” lever than on the “inactive” lever from the 3rd to 14th experimental session [F(13, 234) = 12.66, *p* < 0.001].

The extinction training following cocaine self-administration lasted 10 days; in this phase, neither the drug nor the drug-paired stimuli were given in response to lever pressing, which resulted in a gradual decrease in “active” lever presses. Rats pressed significantly more frequently on the “active” lever than on the “inactive” lever from the 3rd to 19th experimental session [F(23, 414) = 12.08, *p* < 0.001]. As shown in Fig. 2, during the last 3 days of extinction, the total number of “active” lever presses did not differ by more than 10 %.

In the “yoked” cocaine and “yoked” saline groups, the difference between pressing the “active” and the “inactive” lever failed to reach significance (data not shown). The “yoked” cocaine animals received passively exactly the same amount of cocaine (181–191 mg/kg) at the same time as the rats that had learned to actively inject the cocaine.

### Biochemical Studies

#### Cysteine

**Fig. 3:**
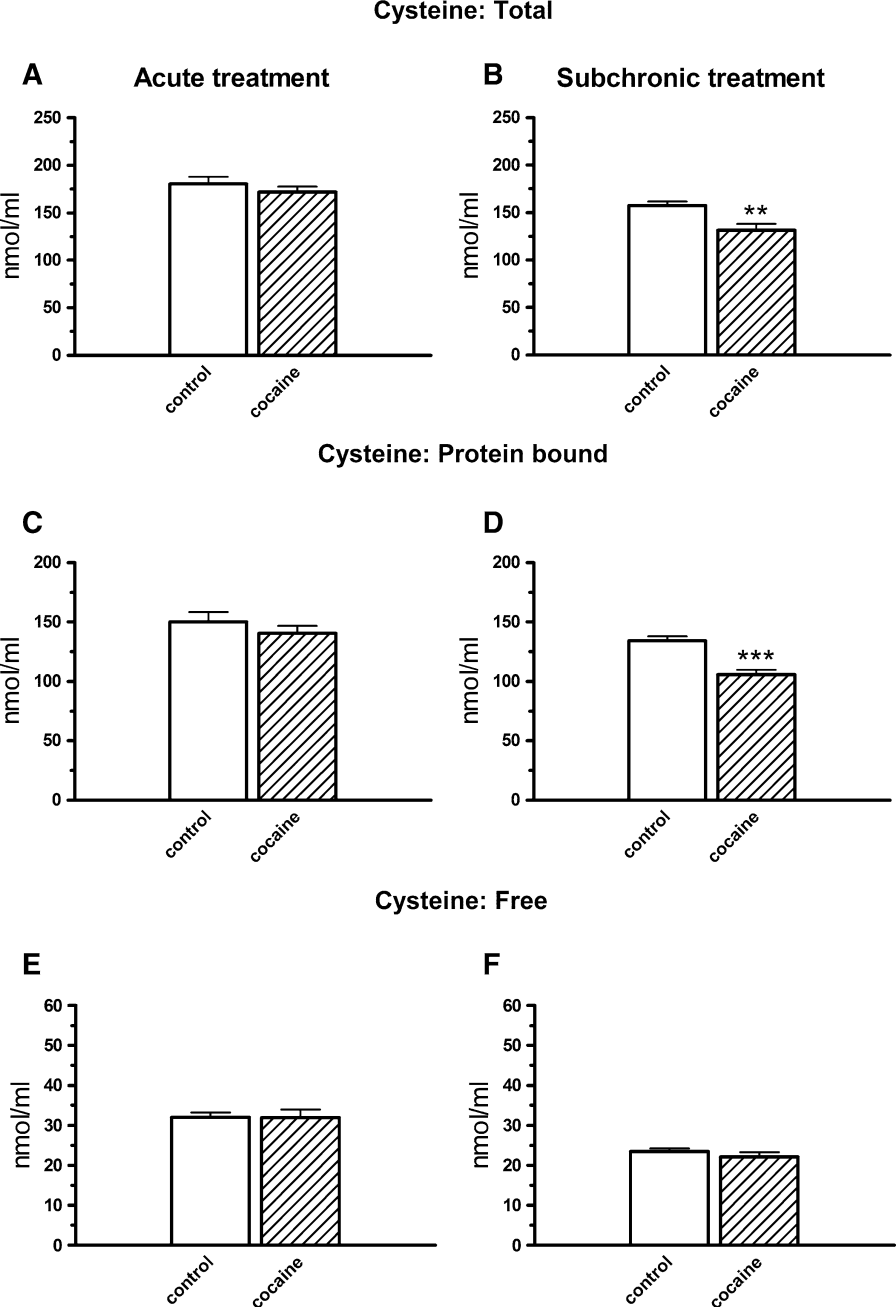
The effects of acute (**a**, **c**, **e**) and subchronic (**b**, **d**, **f**) i.p. treatment with cocaine (10 mg/kg) on the total, protein-bound, and free cysteine levels in the rat plasma. Concentrations of all the cysteine fractions were expressed in nmol/ml, data are presented as the mean ± SEM; *** *p* < 0.001, ** *p* < 0.01 versus control group. The number of animals in experimental groups: acute treatment—control, cocaine—eight rats per group; subchronic treatment—control, cocaine—nine rats per group

Acute i.p. cocaine administration caused no changes in the Cys redox forms under analysis (total Cys: *t* = 0.909, df = 14; protein-bound Cys: *t* = 0.358, df = 14; free Cys: *t* = 0.042, df = 14; *p* > 0.05; Fig. 3a, c, e). However, when cocaine was administered subchronically i.p., the total (*t* = 3.278, df = 16 *p* < 0.01) and protein-bound (*t* = 5.019, df = 16, *p* < 0.001) Cys concentrations markedly decreased (Fig. 3b, d).

**Fig. 4:**
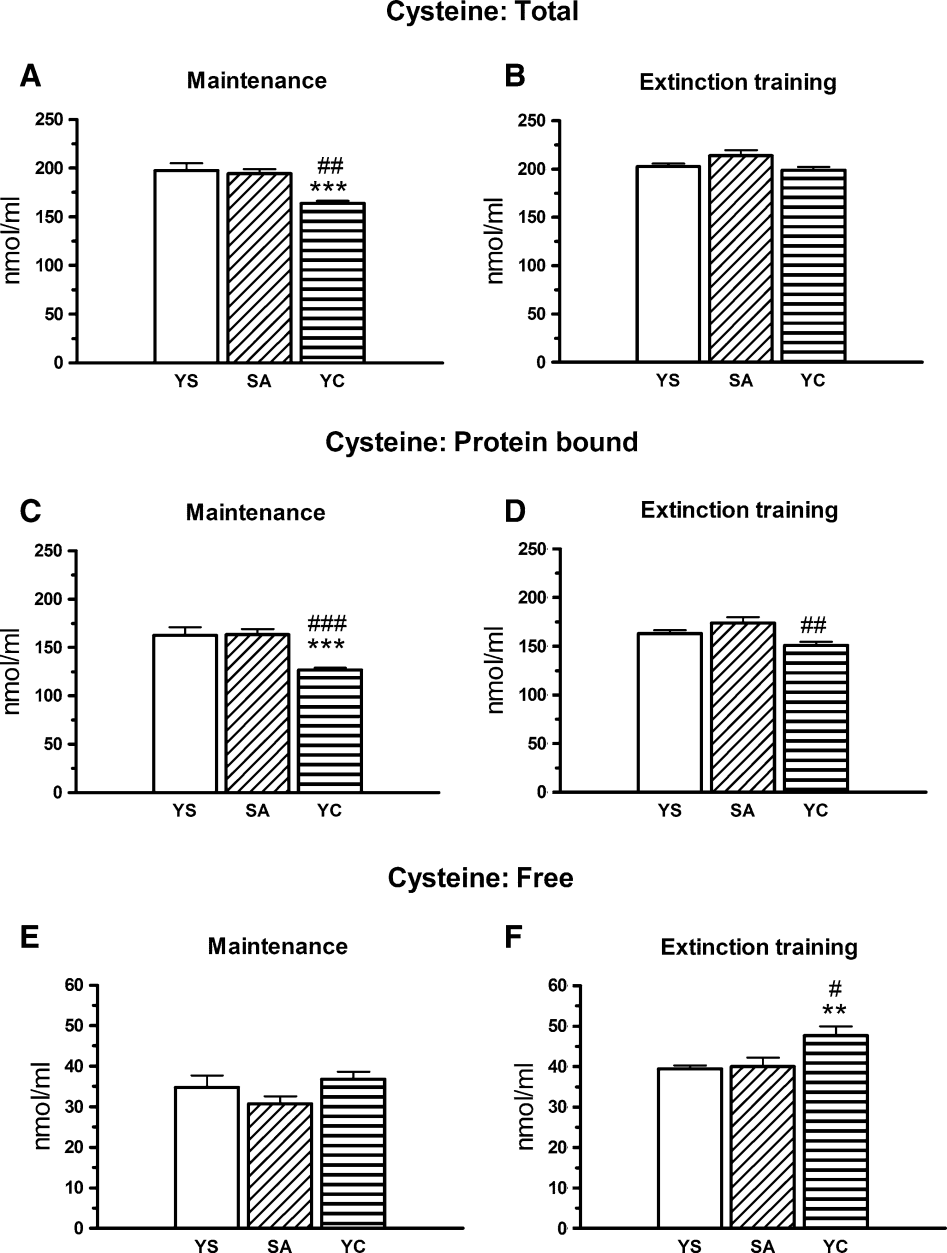
Plasma concentrations of the total, protein-bound, and free cysteine fractions in rats self-administering cocaine (SA) and in the group receiving passive infusions of cocaine (yoked cocaine, YC) at maintenance (**a**, **c**, **e**) and during extinction training (**b**, **d**, **f**). Concentrations of all the cysteine fractions were expressed in nmol/ml, data are presented as the mean ± SEM; *** *p* < 0.001 versus yoked saline (YS), ### *p* < 0.001, ## *p* < 0.01 versus SA group. The number of animals in experimental groups: maintenance—yoked saline (YS)—ten rats, SA—nine rats, YC—ten rats; extinction training: YS—ten rats, SA—nine rats, YC—eight rats

One-way ANOVA revealed a significant effect of treatment on the total [F(2, 26) = 10.862, *p* < 0.001; Fig. 4a] and protein-bound [F(2, 26) = 11.753, *p* < 0.001; Fig. 4c] but not free Cys concentrations [F(2, 26) = 1.753, *p* > 0.05, Fig. 4e] during the maintenance phase. Only in YC rats, there was a significant decrease in the concentrations of the total (Fig. 4a) and protein-bound (Fig. 4c) Cys fractions when compared to YS control (*p* < 0.001) and self-administration group (*p* < 0.01–0.001) while the free Cys fraction remained at the control level (Fig. 4e). No significant changes in the examined Cys fractions were observed during the maintenance in cocaine self-administration group when compared to YS control group.

During the extinction training (no delivery of cocaine), a one-way ANOVA showed a significant effect of treatment on the protein-bound [F(2, 24) = 5.707, *p* < 0.01; Fig. 4d] and free Cys [F(2, 24) = 5.775, *p* < 0.01, Fig. 4f] but not on the total Cys concentration [F(2, 24) = 3.063, *p* = 0.065; Fig. 4b]. In YC rats, the protein-bound Cys concentration was markedly lower than in the self-administration group (*p* < 0.01; Fig. 4d). On the other hand, free Cys fraction was significantly increased when compared to YS control (*p* < 0.01) or the self-administration group (*p* < 0.05; Fig. 4f).

#### Homocysteine

**Fig. 5:**
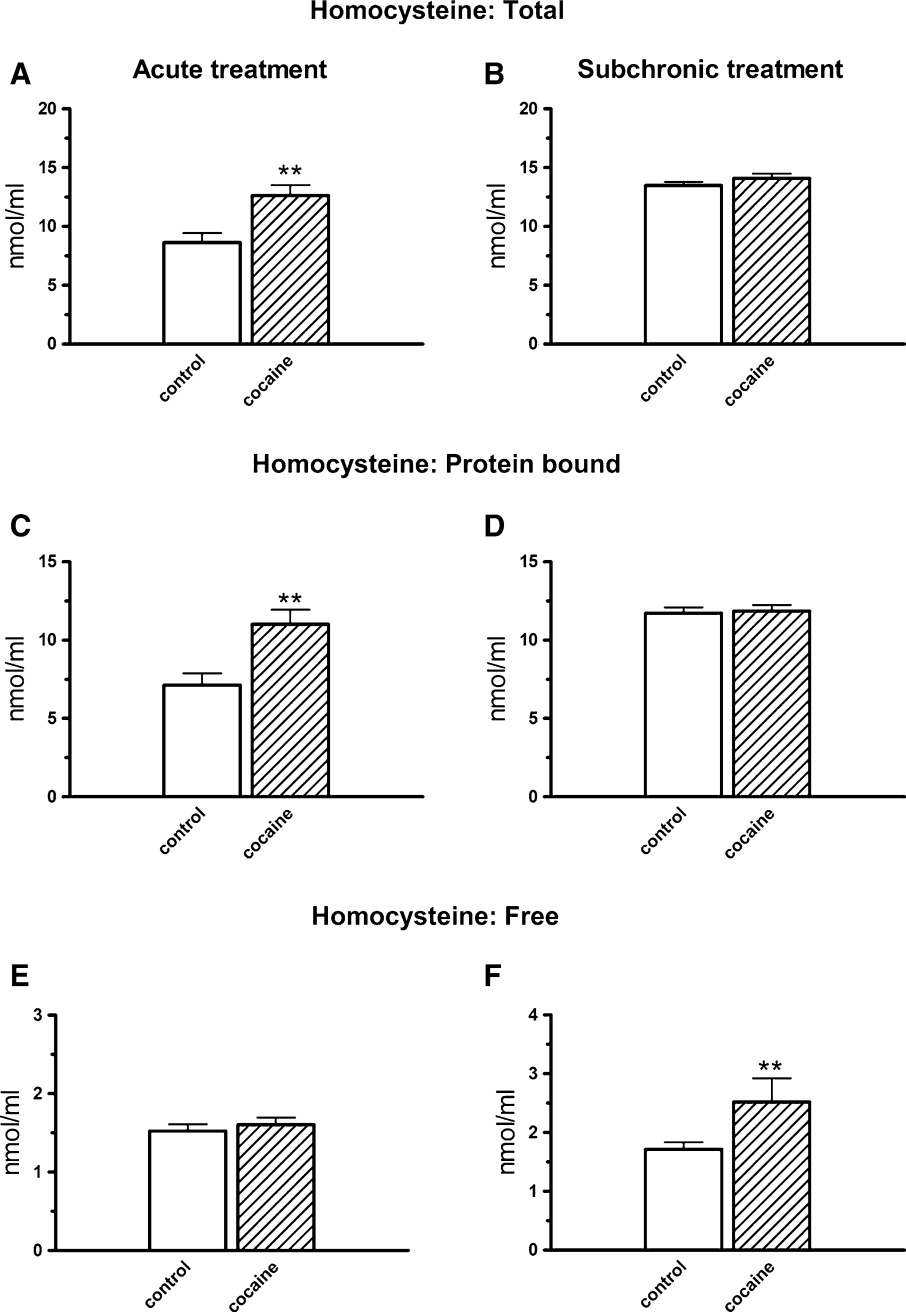
The effects of acute (**a**, **c**, **e**) and subchronic (**b**, **d**, **f**) i.p. treatment with cocaine (10 mg/kg) on the total, protein-bound, and free homocysteine levels in the rat plasma. Concentrations of all the homocysteine fractions were expressed in nmol/ml, data are presented as the mean ± SEM; *** *p* < 0.001, ** *p* < 0.01 versus control group. The number of animals in experimental groups: acute treatment—control, cocaine—eight rats per group; subchronic treatment—control, cocaine—nine rats per group

Acute cocaine treatment increased the total (*t* = −3.411, df = 14, *p* < 0.01) and protein-bound (*t* = −3.319, df = 14, *p* < 0.01) Hcy fractions (Fig. 5a, c) while free fraction was unchanged (*t* = −0.658, df = 14, *p* > 0.05; Fig. 5e). In opposite, when cocaine was injected subchronically i.p., the concentrations of total (*t* = 1.175, df = 16, *p* > 0.05) and protein-bound Hcy (*t* = −0.226, df = 16, *p* > 0.05; Fig. 5b, d) remained unchanged while the free fraction significantly (*t* = −3.566, df = 16, *p* < 0.01) increased (Fig. 5f).

**Fig. 6:**
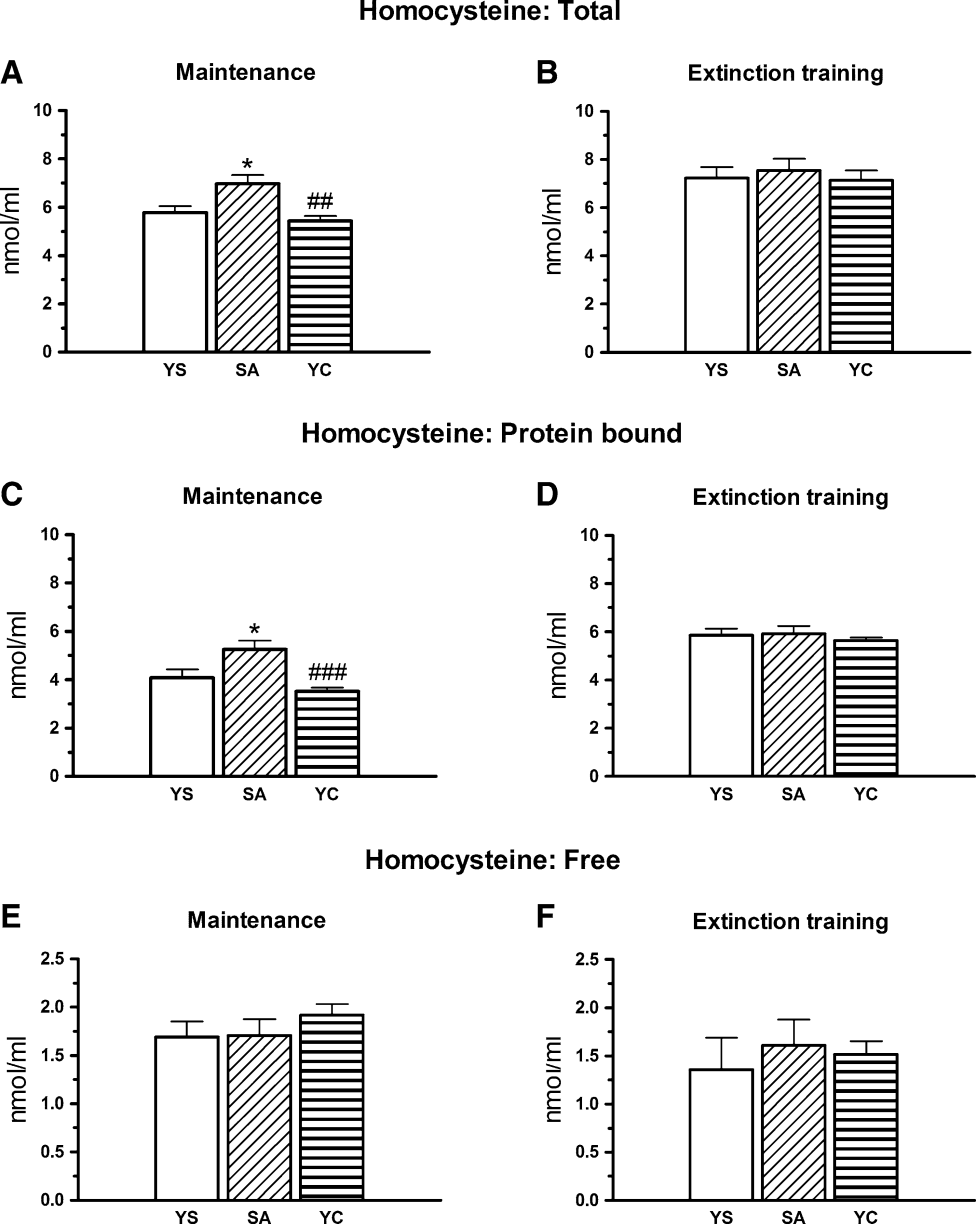
Plasma concentrations of the total, protein-bound, and free homocysteine fractions in rats self-administering cocaine (SA), and in the group receiving passive infusions of cocaine (yoked cocaine, YC) at maintenance (**a**, **c**, **e**) and during extinction training (**b**, **d**, **f**). Concentrations of all the homocysteine fractions were expressed in nmol/ml, data are presented as the mean ± SEM; ** *p* < 0.01 versus yoked saline (YS), ### *p* < 0.001 versus SA group. The number of animals in experimental groups: maintenance—yoked saline (YS)—ten rats, SA—nine rats, YC—ten rats; extinction training: YS—ten rats, SA—nine rats, YC—eight rats

A one-way ANOVA showed a significant treatment effect on the total [F(2, 26) = 8.120, *p* < 0.002; Fig. 6a] and protein-bound [F(2, 26) = 9.368, *p* < 0.001; Fig. 6c] but not free Hcy concentrations [F(2, 26) = 0.759, *p* > 0.05, Fig. 6e] during the maintenance. In the cocaine self-administration group, the total and protein-bound Hcy fractions were significantly increased when compared to the YS control (*p* < 0.05; Fig. 6a, c) whereas the free fraction remained at the control level (Fig. 6e).

During the extinction procedure in rats previously administered cocaine, a one-way ANOVA revealed a lack of significant treatment effect on concentrations of the total [F(2, 24) = 0.210, *p* > 0.05, Fig. 6b], protein-bound [F(2, 24) = 0.239, *p* > 0.05, Fig. 6d], and free [F(2, 24) = 0.136, *p* > 0.05; Fig. 6f] Hcy fractions. Interestingly, in the YC group, no significant changes in any Hcy fraction were seen either during treatment (Fig. 6a, c, e) or after the 10-daily extinction training (Fig. 6b, d, f).

Thus, a single acute cocaine treatment and cocaine self-administration induced similar changes in concentration of the total and protein-bound Hcy fractions.

#### Labile, Reduced Sulfur

**Fig. 7:**
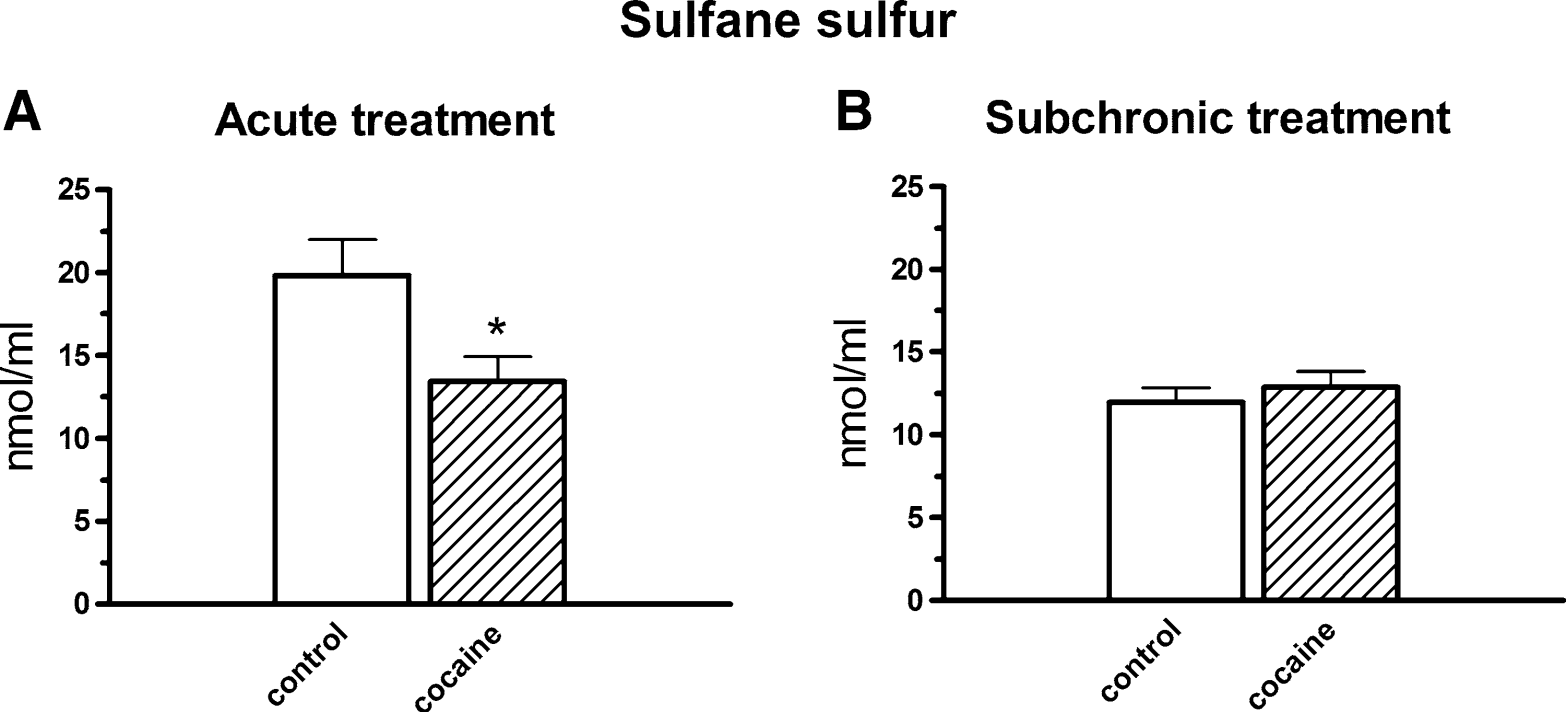
The effects of acute (**a**) and subchronic (**b**) i.p. treatment with cocaine (10 mg/kg) on the levels of sulfane sulfur in the rat plasma. Concentrations of sulfane sulfur were expressed in nmol/ml, data are presented as the mean ± SEM; * *p* < 0.05 versus control group. The number of animals in experimental groups: acute treatment—control, cocaine—eight rats per group; subchronic treatment—control, cocaine—nine rats per group

After acute cocaine (i.p.) treatment, the LS level markedly decreased (*t* = 2.426, df = 14, *p* < 0.05; Fig. 7a) while chronic drug administration did not evoke the changes in its level (*t* = −0.713, df = 14, *p* > 0.05; Fig. 7b).

**Fig. 8:**
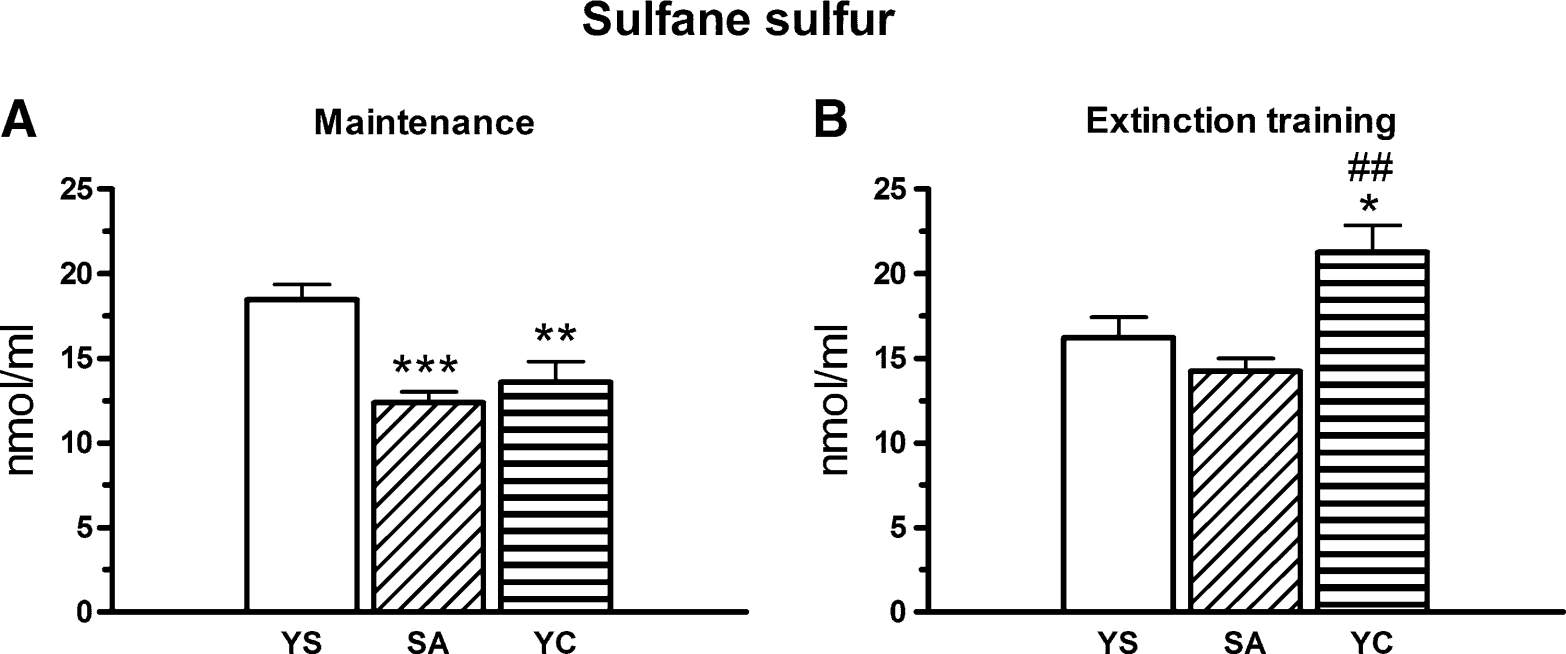
Plasma concentration of sulfane sulfur in rats self-administering cocaine (SA), and in the group receiving passive infusions of cocaine (yoked cocaine, YC) at maintenance (**a**) and during extinction training (**b**). Concentration of sulfane sulfur was expressed in nmol/ml, data are presented as the mean ± SEM; *** *p* < 0.001 versus yoked saline (YS), ### *p* < 0.001 versus SA group. The number of animals in experimental groups: maintenance—yoked saline (YS)—ten rats, SA—nine rats, YC—ten rats; extinction training: YS—ten rats, SA—ten rats, YC—ten rats

A one-way ANOVA showed a significant treatment effect on plasma concentrations of LS during the maintenance [F(2, 26) = 10.836, *p* < 0.001; Fig. 8a] and extinction training [F(2, 27) = 8.682, *p* < 0.002; Fig. 8b]. During the maintenance, LS content was decreased both in the cocaine self-administered (*p* < 0.001) and YC groups (*p* < 0.01) when compared to YS control (Fig. 8a). It means that drug operant is responding lowered LS level independently of the way of cocaine administration. Diverse responses were observed after the extinction training since LS level returned to the control values in the cocaine self-administration group while in the YC group it was significantly enhanced when compared to YS control (*p* < 0.02) or self-administration group (*p* < 0.002; Fig. 8b).

## Discussion

The present studies indicated for the first time that cocaine treatment significantly altered plasma concentrations of different redox forms of Cys and Hcy that were dependent on the route and manner (voluntary vs. passive) of cocaine administration. Moreover, some long-lasting changes in the contents of these sulfur-containing amino acids were also observed during extinction training in drug-free period. Consequently, our experiments demonstrated that either cocaine self-administration or its acute i.p. treatment resulted in the increased plasma concentrations of the total and protein-bound Hcy. However, these increases reached the control level after 10-day extinction training in animals self-administering cocaine previously. Since such increases in the total and protein-bound Hcy levels are characteristic of homocysteinemia, our data may indicate that cocaine caused homocysteinemia during self-administration (modeling rewarding properties of cocaine) and after acute treatment. Interestingly, no increases in any redox forms of Hcy were seen in the YC group while in the group subchronically i.p. treated with cocaine the free Hcy concentration increased.

**Fig. 9:**
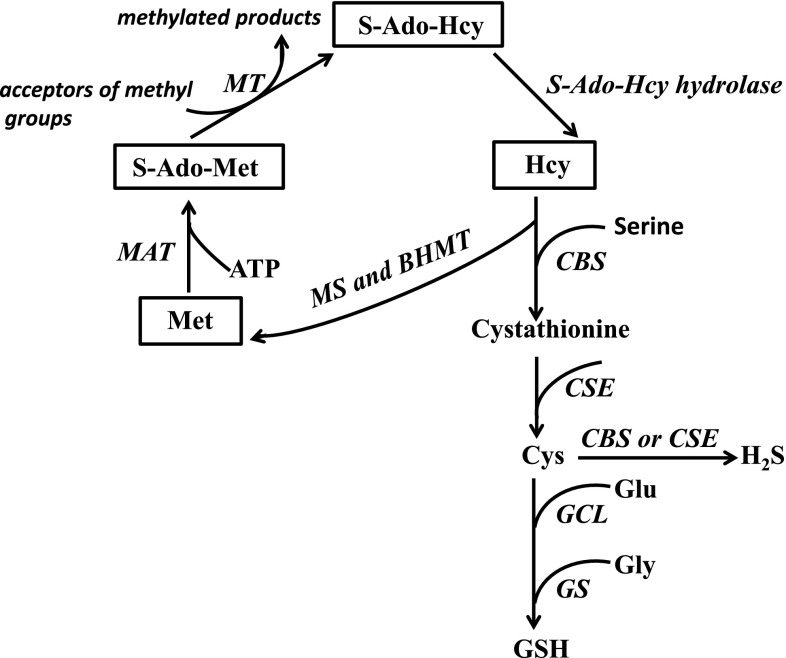
Schematic representation of methionine metabolism via methionine cycle and transsulfuration pathway showing cysteine as a precursor of GSH and hydrogen sulfide (H_2_S). Dietary Met is activated by conversion to S-adenosylmethionine (S-AdoMet also termed SAM) in an ATP-dependent reaction catalyzed by methionine adenosyltransferase (MAT). Then, in the transmethylation pathway S-AdoMet donates its methyl group to a large variety of acceptors (DNA, RNA, histones, phospholipids) through the action of different methyltransferases (MTs) yielding S-adenosylhomocysteine (S-AdoHcy also termed SAH) that is hydrolyzed to form Hcy and adenosine via a reversible reaction catalyzed by S-AdoHcy hydrolase. S-AdoHcy is a potent competitive inhibitor of methylation reactions, therefore, the fast removal of Hcy and adenosine is required to prevent accumulation of S-AdoHcy. Hence, Hcy is either re-methylated back to Met using the methyl group provided by 5-methyltetrahydrofolate or irreversibly converted into Cys via transsulfuration pathway. Re-methylation of Hcy is catalyzed by methionine synthase (MS) or by betaine Hcy methyltransferase (BHMT). The first of these enzymes which is expressed in all mammalian tissues requires normal level of folate and vitamin B_12_ while the second one is confined to the liver and kidney and requires the presence of betaine, a metabolite of choline. In hepatic cells in particular, dietary Cys acts in methionine-sparing capacity and promotes re-methylation of Hcy to Met. However, when the supply of Cys is insufficient, Hcy in the liver as well as in the brain astrocytes is channeled into the transsulfuration pathway (Vitvitsky et al. 2006; Lu 2009; Kandil et al. 2010; McBean 2012). Thus, in the reaction catalyzed by B_6_-dependent enzyme cystathionine β synthase (CBS), Hcy condenses with serine to form cystathionine which in the reaction mediated by cystathionine γ lyase (CSE termed also CTH) releases free Cys that is used for GSH synthesis. Two enzymes are involved in the latter reaction: γ-glutamate cysteine ligase (GCL) and glutathione synthase (GS). *Abbreviations*: *BHMT* betaine homocysteine methyltransferase, *CBS* cystathionine-β-synthase, *CSE* cystathionine-γ-lyase, *GCL* γ-glutamate cysteine ligase, *Glu* glutamate, *Gly* glycine, *GS* glutathione synthase, *MAT* methionine adenosyltransferase, *MS* methionine synthase, *MT* methyltransferase

Hcy is a sulfur-containing amino acid generated during methionine (Met) metabolism (Banerjee and Zou 2005; Lu 2009) that due to bearing a highly reactive sulfhydryl group easily react with other molecules. Mechanisms involved in the cocaine-induced increases in the total and protein-bound plasma Hcy levels described in the present study are unknown. However, considering possible pathways of Hcy metabolism presented in Fig. 9, it is reasonable to assume that disturbances in Hcy re-methylation to Met and/or its transsulfuration to Cys may play an important role here because both these reactions maintain plasma and cellular levels of Hcy under control. Re-methylation of Hcy to Met occurs in most cells of the body while its transsulfuration to Cys occurs only in the liver, pancreas, kidney, small intestine (Githens 1991) and as recently demonstrated in brain astrocytes (Vitvitsky et al. 2006; Kandil et al. 2010; McBean 2012). Normal levels of cobalamin (vitamin B_12_) and folate are essential cofactors limiting re-methylation of Hcy while vitamin B_6_ is a cofactor limiting its transsulfuration (Fig. 9). Inhibition of Hcy re-methylation to Met may lead to a decline of S-AdoMet content, the primary methyl group donor that plays the central role in many biological processes also in gene expression via DNA methylation (Mato et al. 2002; Kim 2005). In line with the latter fact, it is worth to mention that a decreased DNA methylation was reported to follow repeated cocaine exposure (Tian et al. 2012), and dysregulation of DNA methylation was suggested to be related with cocaine addiction (Kim 2005).

On the other hand, the inhibition of Hcy transsulfuration pathway can cause a decline of Cys level that is a limiting factor for GSH synthesis (Beatty and Reed 1980). In line with this suggestion, it has been demonstrated that cocaine decreased the plasma concentration of GSH (Labib et al. 2001, 2002; Visalli et al. 2005), and the latter effect was attributed to the increased production of ROS by cocaine (Dietrich et al. 2005; Kovacic 2005; Visalli et al. 2005). In the present study, in rats receiving YC infusions and in those treated subchronically i.p. with this drug of abuse, the plasma levels of total and protein-bound Cys were markedly decreased but the content of free Cys was maintained at the control level (Table 1). Simultaneously, such subchronic i.p. cocaine administration increased the level of free Hcy while its two other fractions remind unchanged. In contrast to YC animals and those given subchronically i.p. drug injections, in rats self-administering cocaine in which the total and protein-bound Hcy levels were significantly enhanced while the free Hcy content was maintained at the control level, there were no changes in the levels of the examined redox forms of Cys (Table 1). The latter findings suggest the triggering compensatory mechanisms under conditions of cocaine self-administration that prevented the decline of plasma Cys concentration which is a very important redox regulator (Kemp et al. 2008; Jones et al. 2004). It is hypothesized that paradoxical activation of Hcy transsulfuration in the liver and/or the increased GSH degradation to constituent amino acids in the kidney of rats voluntary administering cocaine, could be the mechanisms that keep plasma Cys concentration at the control level. Consistently with this assumption, an increase in GSH content reported in the liver of cocaine-treated rats (Wiener and Reith 1990; Mehanny and Abdel-Rahman 1991; Labib et al. 2001, 2002) seems to indicate the activation of transsulfuration pathway. However, since the increases in the total and protein-bound Hcy levels were observed also 1 h after acute i.p. cocaine administration, in the absence of changes in the content of the examined Cys redox forms (Table 1), it was assumed that the Hcy could also displace Cys from other protein-bound thiol molecules. Further studies are required to explain all the above discrepancies and the impact of cocaine on the Hcy metabolism.

**Table 1:** The effects of cocaine on the levels of different redox forms of homocysteine, cysteine and labile, reduced sulfur in the rat plasma following active versus passive drug injections

Treatment	Homocysteine			Cysteine			Labile, reduced sulfur (LS)
	Total	Protein-bound	Free	Total	Protein-bound	Free	
Maintenance							
Cocaine (0.5 mg/kg/infusion) self-administration (SA)	**↑**	**↑**	**–**	**–**	**–**	**–**	**↓**
Cocaine (0.5 mg/kg/infusion) yoked (YC)	**–**	**–**	**–**	**↓**	**↓**	**–**	**↓**
Extinction training							
Cocaine (0.5 mg/kg/infusion) self-administration (SA)	**–**	**–**	**–**	**–**	**–**	**–**	**–**
Cocaine (0.5 mg/kg/infusion) yoked (YC)	**–**	**–**	**–**	**–**	**–**	**↑**	**↑**
Acute systemic treatment							
Cocaine (10 mg/kg, i.p.)	**↑**	**↑**	**–**	**–**	**–**	**–**	**↓**
Subchronic systemic treatment							
Cocaine (10 mg/kg, i.p.) for 5 days	**–**	**–**	**↑**	**↓**	**↓**	**–**	**–**

**↑** increase; **↓** decrease; – lack of changes

Hcy is a commonly accepted independent risk factor of atherosclerosis and thrombotic complications (Refsum et al. 1998). Thus, the increased plasma total Hcy level in rats self-administering cocaine may indicate an increased risk of atherosclerosis and myocardial infarction (MI) due to microvascular spasm. Cocaine use has been associated with both acute and chronic cardiovascular diseases which include acute MI, myocardial ischemia, acceleration of the development of atherosclerosis, and hypertension (Kloner et al. 1992; Rezkalla and Kloner 2007). According to the theory of “small vessel disease” proposed by Tambe (Tambe et al. 1972), SCF is a cause of microvascular spasm. Myocardial microvessels, due to their well-developed muscular layer and small diameters, are significant regulators of coronary flow and the main physiological determinants of the total coronary resistance. MI occurs when one or more of the coronary arteries supplying blood to the heart are occluded depriving a part of the heart of oxygenated blood and nutrients leading to necrosis of the myocardium. Acute MI is the most prevalent form of cardiovascular death. It was also reported to occur in cocaine addicts with normal epicardial arteries and with a low risk of cardiovascular disease (Rezkalla and Kloner 2007; Turhan et al. 2007). Cocaine evokes vasoconstriction primarily by blocking the presynaptic uptake of norepinephrine, and by stimulating postsynaptic α-adrenergic receptors, with a subsequent increase in the calcium flux. Cocaine-induced vasoconstriction leads to an increase in blood pressure and coronary resistance. Also increased platelet aggregability after cocaine can contribute to MI.

On the other hand, the mechanism by which the elevated total Hcy plasma concentration contributes to atherosclerosis has not been completely elucidated, as yet. However, as global DNA hypomethylation has been observed in atherosclerotic lesions in humans and in animal models as a consequence of the elevated Hcy or low-dietary folate concentrations (Castro et al. 2003; Lund et al. 2004; Zaina et al. 2005), it is reasonable to assume that cocaine-induced disturbances in Hcy metabolism indirectly affecting the DNA methylation could contribute to the accelerated atherosclerosis. Additionally, the increased total plasma Hcy accelerates the ROS generation which results in vascular endothelium dysfunction and is one of the early events in atherosclerosis progression (McCully 1996).

In the light of the above considerations, the question arises whether the increased concentration of the Hcy-protein mixed disulfides may have further implications. In fact, protein-thiol mixed disulfides are formed in a reversible reaction of S-thiolation which is thought to be a regulatory and antioxidant mechanism (Włodek and Iciek 2003; Dalle-Donne et al. 2007, 2008; Mieyal et al. 2008). Protein binding of thiol molecules to form mixed disulfides protects protein −SH groups against irreversible oxidation to −SO_2_H and SO_3_H. These mixed disulfides can be formed with different LMW thiols, such as GSH, Cys, or Hcy, of which the latter two are a subject of the present research. Protein S-glutathionylation, the reversible formation of mixed disulfides between glutathione and low-pKa cysteinyl residues of proteins, is an important mechanism for the dynamic, posttranslational modification of a variety of regulatory, structural, and metabolic proteins as well as for the regulation of signaling and metabolic pathways (Dalle-Donne et al. 2007, 2008; Mieyal et al. 2008). A number of proteins known to be affected by cocaine (actin, JNK, nuclear kinase kappa B, cyclic AMP-dependent protein kinase; (Hyman et al. 2006; Kalivas and O’Brien 2008) are regulated by S-glutathionylation (Klatt and Lamas 2002; Humphries et al. 2005; Fiaschi et al. 2006; Reynaert et al. 2006). Based on the above-mentioned studies and the fact that cocaine increased the protein S-glutathionylation in rats, Uys et al. (2011) have recently postulated that signaling associated with this modification may be a key factor of neuroadaptations evoked by this drug of abuse.

Considering the results obtained in the present study, it is worth noting that the formation of the protein-thiol mixed disulfides is determined by characteristics of the protein undergoing S-thiolation, i.e., albumin in plasma. The −SH group is located in hydrophobic environment of the plasma albumin molecule, thus, it is characterized by a much lower pKa (~ 5) than plasma LMW thiols (Carter and Ho 1994). In consequence, at the physiological pH, it is to a greater degree dissociated to form a highly reactive thiolate anion (Alb-S−). As the result of that, about 1/3 of the plasma albumin −SH groups are covalently modified forming albumin-LMW thiol mixed disulfides. For this reason, the albumin is believed to be a transport protein for thiols in the circulation (Sengupta et al. 2001). A greater tendency of Hcy than Cys to form albumin mixed disulfides is also attributable to a higher lipophilicity of Hcy related to an additional methylene group (−CH_2_) in its structure which can facilitate the reaction with the −SH group in hydrophobic environment of the albumin molecule. It is known that plasma albumin, also S-homocysteinylated albumin, can be transported into vascular endothelial cells by endocytosis (Carter and Ho 1994; Sengupta et al. 2001). Then in consequence of intracellular biodegradation of the endocytozed albumin, Hcy level in endothelial cells may increase (Schnitzer and Oh 1994; Sengupta et al. 2001).

In contrast to Hcy, the concentrations of the total, protein-bound, and free Cys which play crucial and independent roles in redox regulatory mechanisms, remain unchanged in the cocaine self-administration group. It means that Cys levels in the cocaine self-administration group are normal, and thus the Cys-related redox potential and regulatory function of this amino acid are preserved. The Cys/CySS redox system is the largest pool of the LMW thiols in plasma. An array of studies confirmed that the changes in the extracellular Cys/CySS ratio affected the most important cellular processes, including the monocyte adhesion to vascular endothelial cells, by influencing the redox potential of plasma and cells (Go and Jones 2005; Sato et al. 2005). Furthermore, yoked infusion of cocaine was accompanied by a drop in the Cys concentration which indicates the changes in redox potential of the most important plasma redox system. In contrast, during extinction training, the free fraction of Cys was increased only in the YC group which suggests the acceleration of Cys autooxidation to CySS.

Regarding cocaine effects on thiol amino acids, their susceptibility to autooxidation yielding ROS-generating disulfides should also be considered. The autooxidation is determined by the pK value of the −SH group, i.e., by the degree of its dissociation to a nucleophilic thiolate anion (−S^−^) (Lash and Janes 1985; Sengupta et al. 2001). Cys and Hcy pK_a_ values are pK_aCys_ = 8.3 and pKa_aHcy_ = 8.87, respectively. Based on the pK_a_ value of −SH group, the ratio of the number of thiol molecules dissociated to thiolate anions RS^−^ to the number of undissociated thiol molecules under physiological conditions (pH 7.4) can be calculated from the following formula:

$$ {\text{RS}}^{ - } /{\text{RSH }} = 10^{{\left( {\text{pH - pKa}} \right)}} $$

The respective values of the above ratio were estimated for CysS^−^/CysSH at 10^(7,4–8,3)^ = 0.126, and for HcyS^−^/HcySH at 10^(7,4–8,87)^ = 0.034. The greater the RS^−^/RSH ratio, the greater concentration of thiolate anions −S^−^, and thus the higher the risk of autooxidative ROS generation. It means that Cys shows a greater tendency to undergo autooxidation than Hcy.

Aerobic Cys metabolism yields sulfates and taurine while its anaerobic metabolism leads to a pool of compounds bearing the LS, which is an H_2_S precursor (Chen et al. 2007; Toohey 2011; Fig. 1). The present studies demonstrated for the first time that cocaine decreased the plasma sulfane sulfur level both in the cocaine self-administration group and cocaine yoked group, and in the group receiving a single i.p. dose of this drug. It could result from the cocaine-induced blockade of LS transport from cells to plasma or from a greater use of LS to compensate for oxidative stress (Everett et al. 1994). The fact that a single acute cocaine injection lowered plasma LS level suggested its participation in the antioxidant defense. After 10-day extinction training sessions in the group previously administered cocaine, LS level returned to the control values but statistically significant increase was achieved only in the YC group. Thus, again there was a difference in the cocaine effect between the active versus YC administration also with respect to the LS level. Conversely, five daily cocaine i.p. treatments did not elicit any statistically significant changes in the LS level.

In the present study of abuse and addiction mechanisms, we incorporated different means of cocaine intake to mimic the typical pattern of drug exposure in laboratory animals. The differences in homeostasis of thiol amino acids Cys and Hcy, and some products of anaerobic Cys metabolism, may be attributable to variations in cocaine serum levels after i.p. versus i.v. drug treatment; and/or related to the regimen of drug dosage, including onset of drug action and duration of effect. Other variables impacting on the outcome relate to the enzymatic degradation, as well as experience of animals (being either drug-naïve or drug-treated). Finally, stress is an inherent complication for yoked animals as well as those given passive i.p. cocaine injections. These aversive procedures can reduce the motivational aspect of cocaine (Twining et al. 2009), and also enhance the corticosterone levels while activating the sympathetic-adrenergic system. Conversely, the increased cocaine seeking behavior in the self-administering group may be linked to reduced food consumption which constitutes the major source of sulfur containing amino acids, particularly methionine and Cys. Hence, not only different routes of the cocaine administration do affect homeostasis of the studied thiol amino acids, but aversive and motivational factors play a part as well.

In conclusion, the present studies indicate that the increase in the total and protein-bound Hcy fraction in the cocaine self-administering group, and after acute treatment was not accompanied by any changes in Cys concentration. In contrast, the following experimenter delivered cocaine (i.p. or i.v.), the total and protein-bound Cys fraction decreased but there were no changes in Hcy concentration. During the extinction training in the group previously administered cocaine, the concentrations of Hcy fractions returned to the control level, whereas YC infusions resulted in an increase in the free Cys fraction, suggesting the occurrence of autooxidation processes in that interval. We also report that the plasma level of reduced LS was lowered by cocaine in the self-administration group and after yoked and acute i.p. cocaine treatment, which suggests a pro-oxidant action of the drug. During extinction training, the LS level regained normal values in the cocaine self-administration group, while LS was significantly increased in animals receiving the YC infusions. Some similarities in the cocaine effects were noted between yoked drug infusions and chronic i.p. treatment and between active cocaine intake and acute cocaine treatment. In summary, our data indicate that the changes in homeostasis of thiol amino acids Cys and Hcy, and some products of anaerobic Cys metabolism are the consequences of the manner in which the drug is administered. These findings provide a better understanding of the use/abuse liability of cocaine.

## Abbreviations

Cys: Cysteine
CySS: Cystine
Hcy: Homocysteine
LS: Labile, reduced sulfur
YC: Yoked cocaine
YS: Yoked saline
